# Raman Spectroscopic and Sensory Evaluation of Cocoa Liquor Prepared with Ecuadorian Cocoa Beans Treated with Gamma Irradiation or Induced Electromagnetic Field Fermentation

**DOI:** 10.3390/foods12213924

**Published:** 2023-10-26

**Authors:** Tania María Guzmán-Armenteros, Jenny Ruales, Cristina Cuesta-Plúa, Juan Bravo, Marco Sinche, Edwin Vera, Edison Vera, Paul Vargas-Jentzsch, Valerian Ciobotă, Fernando E. Ortega-Ojeda, Andrés Proaño, Armando Echeverría, Luis Ramos-Guerrero

**Affiliations:** 1 Departamento de Ciencia de Alimentos y Biotecnología, Facultad de Ingeniería Química y Agroindustria, Escuela Politécnica Nacional (EPN), Quito 170525, Ecuador; tania.guzman@epn.edu.ec (T.M.G.-A.); jenny.ruales@epn.edu.ec (J.R.); edwin.vera@epn.edu.ec (E.V.); 2 Agencia de Regulación y Control Fito y Zoosanitario (AGROCALIDAD), Av. Interoceánica km 14 ½, Tumbaco 170184, Ecuador; maria.cuesta@agrocalidad.gob.ec (C.C.-P.); juanitob920@hotmail.com (J.B.); 3 Departamento de Ciencias Nucleares, Facultad de Ingeniería Química y Agroindustria, Escuela Politécnica Nacional, Ladrón de Guevara E11-253, Quito 170525, Ecuador; marco.sinche@epn.edu.ec (M.S.); edison.vera@epn.edu.ec (E.V.); paul.vargas@epn.edu.ec (P.V.-J.); 4 Rigaku Analytical Devices, Inc., 30 Upton Drive, Suite 2, Wilmington, MA 01887, USA; valerian.ciobota@rigaku.com; 5 Departamento de Ciencias de la Computación, Universidad de Alcalá, Ctra. Madrid-Barcelona Km. 33.6, 28871 Alcalá de Henares, Madrid, Spain; fernando.ortega@uah.es; 6 Instituto Universitario de Investigación en Ciencias Policiales (IUICP), Universidad de Alcalá, Libreros 27, 28801 Alcalá de Henares, Madrid, Spain; 7 Programa de Reactivación de Café y Cacao, Ministerio de Agricultura y Ganadería, Av. Eloy Alfaro y Av. Amazonas, Quito 170518, Ecuador; carlos.proanio@rikolto.org; 8 Facultad de Ciencias Técnicas, Universidad Internacional del Ecuador, Quito 170411, Ecuador; neecheverriall@uide.edu.ec; 9 Grupo de Investigación Bio-Quimioinformática, Carrera de Ingeniería Agroindustrial, Facultad de Ingeniería y Ciencias Aplicadas, Universidad de Las Américas (UDLA), Quito 170503, Ecuador

**Keywords:** chocolate, cocoa beans, cocoa liquor, Raman spectroscopy, magnetic field, fermentation

## Abstract

Cocoa liquor is the primary precursor of the worldwide highly appreciated commodity chocolate. Its quality depends on several factors, such as the type of cocoa, the fermentation process, and the control of the contaminants in the fermented beans. This study aims to evaluate whether the induced magnetic field treatment during the fermentation process or the pathogen reduction with gamma irradiation after the fermentation affect the characteristics of the cocoa liquor obtained from Ecuadorian cocoa beans. For this purpose, liquor samples from controls (standard process), from beans treated with an induced magnetic field up to 80 mT, and from beans irradiated with nominal doses up to 3 kGy were characterized through Raman spectroscopic analysis and sensorial evaluation. The most relevant bands of the cocoa liquor were assigned according to reports from the literature, spectroscopic data, and chemometrics. The spectra corresponding to different treatments and doses were visually very similar, but they could be discriminated using OPLS-DA models, where the most intense Raman signals were attributed to the lipid components. The sensorial evaluation rated the presence of floral, fruity, almondy, acid, and bitter flavors, along with astringency and intense aroma, and these attributes exhibited variable behavior depending on the dose of the irradiation or magnetic treatment. Therefore, both treatments may exert an influence on cocoa beans and, therefore, on the cocoa liquor quality.

## 1. Introduction

The production of cocoa beans and the chocolate industry constitute an essential share of the economy of many countries. Moreover, there has been an increase in consumers’ interest in premium chocolates containing organic, single-origin, and Fairtrade cocoa, as well as chocolates with high cocoa contents [1]. 

Chocolate is a mixture of cocoa liquor, sweeteners, emulsifiers, and other ingredients suspended in cocoa butter or other alternative fat sources [2], subjected to refining, conching, tempering, and standardization [3]. The cocoa fermentation process hugely influences the quality of the chocolate; during this step, the beans go through chemical and physical transformations that define desired characteristics such as aroma and flavor [4]. Cocoa fermentation occurs due to the sequential microbial action of yeasts, lactic acid bacteria and, eventually, acetic acid bacteria [5]. Each group of microorganisms produces specific compounds responsible for the cocoa beans’ sensory and organoleptic attributes. For instance, volatile organic compounds (such as fatty acid ethyl esters and acetate esters) are produced through the action of yeasts involved in reactions of the amino acid metabolism during the fermentation. These esters are responsible for the development of the chocolate’s aromas and flavors characterized by fruity notes [6].

The application of electromagnetic fields can help to improve the fermentation processes of different agricultural products [7], including cocoa beans [8]. This is considered to be a safe technology that does not generate toxic products and is not difficult to apply. Food processes induced by electromagnetic fields have shown effectiveness in terms of bacterial activation, stimulation of their growth, production of metabolites, and improved sensory quality when compared to conventional treatments [9].

On the other hand, consuming chocolate with a high cocoa content has positive effects on health [10], such as lowering blood pressure, inhibiting platelet activation, improving endothelial function, and avoiding insulin resistance [11]. However, these benefits may be overshadowed in chocolate by a microbial attack by, for instance, aerobic psychrotrophic microbes, spores from thermophilic acidophilus, or mesophilic aerobic bacteria [12]; hence, the importance of applying treatments that hamper microorganisms’ growth.

Gamma irradiation has been applied for decades to eliminate food’s microbiological risks (i.e., pathogenic and food-spoilage microorganisms) without compromising nutritional properties, consumer safety, and sensory quality [13]. In this technology, food products are exposed to the ionizing radiation emitted by cobalt-60 or cesium-137 radioisotopes, which have high energy and a penetration capacity of several feet [14]. Additional applications of irradiation of food materials include shelf-life extension, delaying ripening in fresh products [15], sprout inhibition in bulbs and tubers, insect disinfesting in cereals, pathogen elimination or reduction in animal products and vegetables [16], and improving food’s mycotoxicological safety [17]. Food irradiation, also known as ionization, exhibits exciting benefits such as the absence of chemical residues after the treatment, the avoidance of important temperature increases during the process, versatility since it can be applied to low- and high-moisture products as well as fresh or frozen food [18], and easiness regarding the operation of the irradiators, since the gamma rays are emitted in all directions continuously and at a predictable rate [19]. Moreover, the process does not require much electric energy, and it is not a water-consuming method. However, gamma rays are unsuitable for some products, like plums and pears, in which quality properties are affected by the reduction in firmness [20]. The dose sensitivity of a product depends on its composition, because some macromolecules are more susceptible to radiation than others (lipids > carbohydrates > proteins). In this sense, radiation is widely recommended for fish, meat, and poultry. Conversely, fruits and vegetables are characterized by higher contents of carbohydrates that could experience hydrolysis reactions, while foods with high lipid contents are susceptible to auto-oxidation [21]. Therefore, the dose must be carefully selected depending on the nutritional profile of the product.

Food exposed to a proper irradiation dose for technical purposes is safe and nutritionally adequate according to international experts [22]. The flavor and some nutritional properties could undergo modifications, but these changes are generally less noticeable than those observed when applying conventional preservation treatments (e.g., cooking, canning, pickling, freezing, and drying) [17]. Additionally, food ionization could be more expensive than other methods, the availability of irradiation facilities is limited, and certain importing markets decline irradiated products [23]. Nevertheless, the unit costs can be lowered if larger volumes of product are subjected to this treatment, and if irradiation facilities operate close to their maximum capacity. In addition, many products have overcome trade barriers through gamma irradiation [24]. Many people still resist accepting irradiated food due to misconceptions about ionizing radiation. In these cases, consumer reliance on this technology could be acquired through education and scientific evidence. All things considered, gamma treatments exhibit a more significant number of advantages than drawbacks. The key challenge of this food technology is to identify the dose at which the desirable aspects are maximized while shortcomings are minimized.

Another critical subject of concern, in addition to the fermentation process under induced magnetic fields and microbial safety, is that chocolate quality depends on the raw material quality and the manufacturing steps [25]. Physical, chemical, and sensory methods are used to analyze the chocolate quality. Safety, color, aroma, flow behavior, and texture are the principal quality parameters in chocolate [26]. In addition, other quality attributes that are assayed in chocolates include particle size distribution, viscosity, melting profile, and hardness [27], sensory properties related to mouthfeel [28], and fat, protein, carbohydrate, water, and ash contents [29].

At the same time, there is an increasing requirement for quick, objective, and accurate techniques to inspect the quality of foods like chocolate [30]. In this respect, spectroscopic tools (e.g., Raman, IR, etc.) are fast, simple, and inexpensive; they do not require much sample preparation, cause almost no sample destruction, and do not need reagents that later constitute chemical waste [31,32]. In this regard, Raman spectroscopy has a wide range of potential usages in food quality assessment, including the detection of components in the food matrix such as macromolecules (e.g., protein, carbohydrates, fat, etc.), migration of packaging material to the product, the presence of synthetic dyes and carotenoids, determination of changes experienced during stages of manufacture at a structural or conformational level, and the detection and evaluation of counterfeits and adulterations, sample structure, type, molecular conformation, crystallinity, and microorganisms, among many other factors [33,34,35].

In this regard, this study aims to evaluate whether the cocoa bean fermentation process performed under an induced magnetic field and the gamma irradiation of naturally fermented cocoa beans could cause changes in the quality of their respective cocoa liquors. For this purpose, spectroscopic measurements were carried out on cocoa liquor samples from regularly fermented and non-irradiated beans (control), from irradiated beans (up to 3 kGy), and from beans fermented under electromagnetic field treatments (up to 80 mT). At the same time, the impact of gamma irradiation on the sensory profile was also analyzed through a sensory evaluation carried out on non-irradiated and irradiated samples.

## 2. Materials and Methods

### 2.1. Cocoa Bean Fermentation under Induced Magnetic Fields

The CCN-51 cocoa (*Theobroma cacao* L.) pods were obtained from local producers in Santo Domingo de los Tsáchilas in Ecuador. The pods did not have defects or damage, and they had a uniform orange color (an indicator of their degree of maturity). The obtained grains were duly packed in polyethylene bags, transported, and stored (7–12 °C for less than 32 h) in a cold room until the tests. The cocoa pods were washed and opened manually to obtain clean grains without impurities or defects. For each assay, a total of 3 kg of ready-to-ferment slime beans was placed in 5 L plastic fermentation boxes with ventral and lateral openings. The incubation system temperature (37 ± 0.5 °C) and humidity (85 ± 0.8%) were controlled and maintained (B 5042, Heraeus, Hanau, Germany) throughout the fermentation process. The fermentation was stopped after seven days, and the beans were then dried in a forced-air rotary kiln (R610 Roller, Rotary Kiln, Nove, Italy) at 60 °C for 4 h to pass to the next step, as described in Section 2.3.

Subsequently, 24 h after starting the fermentation process, the samples were subjected to a second mild fermentation (in an aerobic phase) under low-frequency magnetic fields of 0 (control), 5, 48, 62, and 80 mT, using a Helmholtz prototype for electromagnetic emissions. The prototype consisted of a designed and validated Helmholtz coil with an area of 0.2 m, an alternating current generator, and a digital signal connection system (to control the uniformity of the oscillating magnetic field (OMF) between the coils). The setup and operation details are available in a previous report [8]. 

### 2.2. Gamma Irradiation Treatment of Fermented Cocoa Beans

The regularly fermented and dried cocoa beans from the National and CCN-51 varieties were maintained at room temperature (18 °C). The cocoa beans (1.3 kg) were packed in polystyrene trays covered with a plastic film, placed vertically, and then irradiated at nominal doses from 0.10 to 3.00 kGy in triplicate (Tables S1 and S2). They were irradiated in a Co-60 panoramic irradiator (Laboratorio de Tecnología de Radiaciones, Escuela Politécnica Nacional, Quito, Ecuador). Non-irradiated samples of each variety were used as controls for the experiment. The dosimetry was assessed by four pellet alanine dosimeters, Batch E2044562 (Bruker BioSpin, Quito, Ecuador), placed in one of the replicates at different tray positions (Figures S1 and S2). The exposed dosimeters were read using an electron paramagnetic resonance spectrometer (e-scan, Bruker BioSpin, Billerica, MA, USA) [36].

### 2.3. Cocoa Liquor Samples

The liquor samples were prepared from CCN-51 cocoa beans via the fermentation process under induced magnetic fields (0, 5, 48, 62, or 80 mT), and from CCN-51 and National cocoa beans after being fermented and irradiated (0.10, 0.20, 0.30, 0.45, 0.60, 0.75, 1.00, 2.00, and 3.00 kGy nominal doses only for sensorial treatment). The cocoa liquor was prepared by an in-house traditional method; for instance, 200 cocoa beans were toasted at 123 °C for 10 min in an oven (UF30, Memmert, Schwabach, Germany). Then, the beans were manually peeled and pre-grinded (KN 295, Foss, Hillerød, Denmark) for 2 min at room temperature (25 °C). The ground beans were refined (502, NCM, Taipei, Taiwan) for 15 min. The obtained cocoa liquor was poured into casts, cooled at 15 °C, and stored in labeled plastic bags at 0 °C once the liquor was solidified.

### 2.4. Raman Spectral Measurements of Cocoa Liquor Samples

Raman measurements of the cocoa liquor samples were performed using a spectrometer (BD_Progeny_LT, Rigaku Raman Technologies, The Woodlands, TX, USA) with a 1064 nm Nd:YAG laser source and a Peltier-cooled InGaAs photodiode array. The temperature was controlled with a cryogenically cooled Ge detector to ensure the solid state of the samples. The measurement conditions were 490 mW, 1000 ms of exposure, and 5 counts. The measurements were taken randomly at different spots on the sample’s surface, and each sample had 20 replicates. Before the Raman measurements, a daily calibration method was carried out with a benzonitrile standard.

### 2.5. Sensorial Evaluation of Cocoa Liquor from Gamma-Irradiated Cocoa Beans

Six trained panelists assessed the sensory profile of the liquors corresponding to the irradiated CCN-51 and National cocoa beans, using a quality rating method. The following attributes were considered: floral, fruity, almondy or nutty, cocoa, acid, bitter, and astringent flavors, as well as aroma intenseness. The parameters were rated in duplicate, with an intensity scale from 0 to 5 (0 = absence; 1 = very mild; 2 = mild; 3 = medium; 4 = strong; 5 = very strong). The average ambient conditions of the testing/sensory room were 25 ± 2 °C and 56% relative humidity. The sensory evaluation was performed similarly to a previously reported method [37] for liquor samples, with some modifications.

A portion of the liquor was placed on the tongue’s surface to identify and quantify the attributes of interest. The panelists rinsed their mouths with water to remove the remaining flavors and waited 5 min before testing another sample. The cocoa liquors were tested in a solid state, at room temperature.

### 2.6. Multivariate Data Analysis of the Raman Spectra of Cocoa Liquor

The Raman spectra were imported in bulk using a script (steps’ sequence) in Origin v2021 9.8 (OriginLab Corporation, Northampton, MA, USA). The spectra were moved and organized sequentially in Excel (Microsoft Corporation, Redmond, USA) and then transposed to have the objects (observations, subjects, or items) in rows and the wavelengths (variables) in columns. Then, the required demographic, categorical, and qualitative variables were added to describe the different samples, groups, categories, or classes.

A few spectra were plotted randomly to identify the spectral zone corresponding to the region of interest (ROI). The rest of the ranges (without information) were removed from the data. The spectral ROI of all samples was then treated with a well-known preprocessing sequence commonly used within spectroscopy. The sequence consisted of a baseline correction, SNV standardization (subtracting the arithmetic mean and dividing by the standard deviation), and soft smoothing (noise reduction) using the Savitzky–Golay method. Finally, the multivariate data analysis was performed using the orthogonal partial least squares regression with discriminant analysis (OPLS-DA) in SIMCA (Sartorius, Göttingen, Germany). This analysis was performed in a procedure that seeks the most significant possible separation between samples.

## 3. Results and Discussion

### 3.1. Cocoa Liquor Raman Spectra

**Figure 1:**
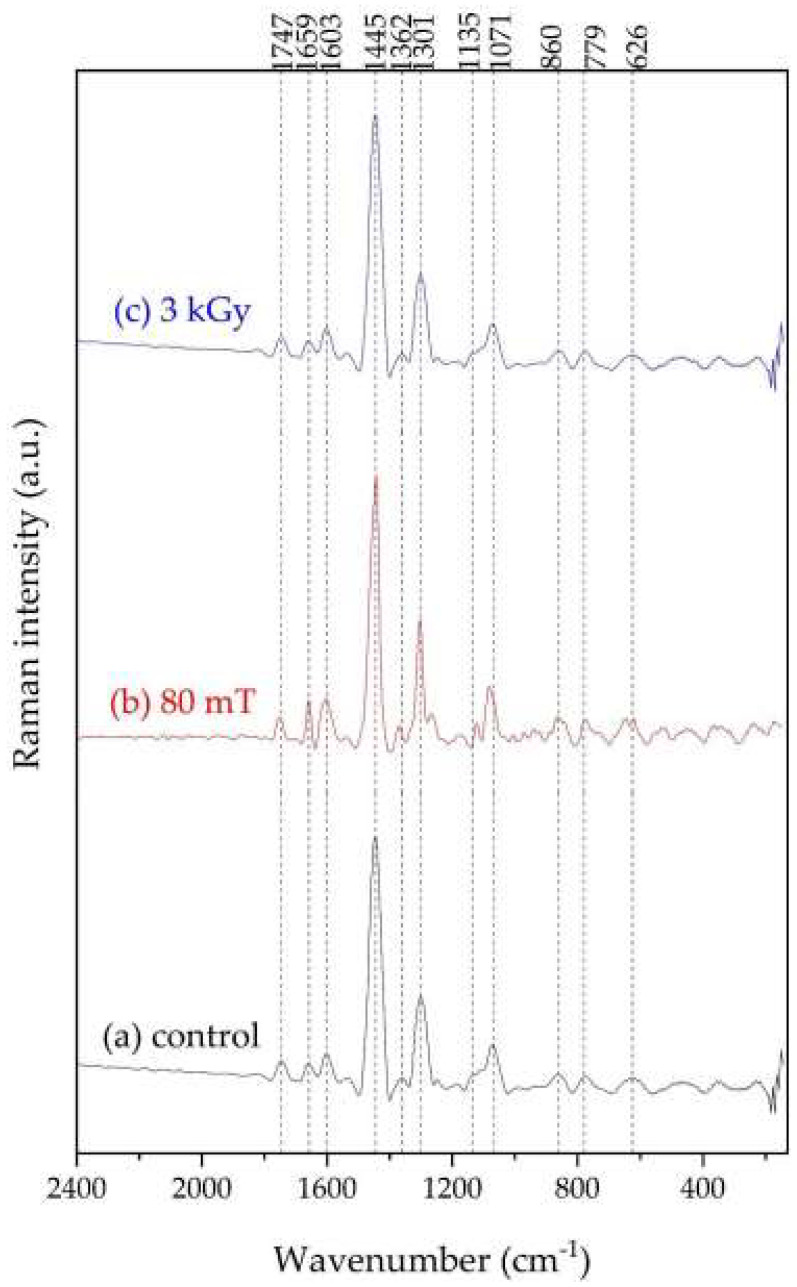
Average Raman spectrum of the cocoa liquor from the CCN-51 variety: (a) standard process (control); (b) fermentation process under induced magnetic field (80 mT); (c) gamma irradiation process (3 kGy).

Figure 1 shows the average Raman spectra of 20 cocoa liquor samples prepared with the CCN-51 variety. Table 1 shows the frequency assignment for the samples shown in Figure 1.

The main constituents of cocoa liquor are lipids, since around 44% of its precursor (cocoa beans) corresponds to this type of molecules [38]. Triacylglycerols (TAGs) account for around 98% of the cocoa lipids, and their composition differs based on geographical origin [39]. Cocoa butter is formed mainly by three TAGs: glycerol-1-palmitate-2-oleate-3-stearate (POS), glycerol-1,3-distearate-2-oleate (SOS), and glycerol-1,3-dipalmitate-2-oleate (POP) [40]. Palmitic (C16:0), stearic (C18:0), oleic (C18:1) [41,42], and linoleic (C18:2) acids [43] are the main components of the TAG cocoa butter fatty acid profile. In this sense, the Raman spectra of the cocoa liquor should also be mainly represented by the spectral signals of those compounds. Indeed, the Raman spectra of cocoa liquor exhibit more intense bands at 1071, 1301, and 1445 cm^−1^, which occur at similar wavelengths found in fatty acid moieties reported previously in the literature, corresponding to the C–C stretching, C–H twisting, and scissoring deformation modes, respectively [44]. Accordingly, the Raman measurements conducted in cocoa butter showed bands regarding the form-V polymorphs at 1063 and 1297 assigned to the stretching v(C–C) and skeletal and τ(CH_2_) twisting modes, respectively [45]. Also, the Raman peaks in several TAGs (i.e., tripalmitin, trimyristin, trilaurin, triundecanin, and triacetin) showed strong bands due to the CH_2_ scissoring vibration at 1445 cm^−1^ [46,47]. Therefore, the mentioned bands of cocoa liquor can be assigned confidently to fatty acid groups and alkanoyl moieties of the cocoa triacylglycerols.

Additionally, it should be noted that most of the Raman bands occurring in lipid materials are ascribed to the acyl chain, which is why the fatty acid spectra are similar to the TAG spectra containing them [48]. Hence, the following bands have been reported: between 1050 and 1150 cm^−1^, corresponding to v(C–C) stretching, at 1296 cm^−1^ for δ(CH_2_) twisting, and between 1400 and 1500 cm^−1^ for δ(CH_3_) or δ(CH_2_) deformations [49]. The literature has indicated that monosaturated and unsaturated fatty acids show peaks occurring at the same wavenumbers, with the difference that the latter have broader bands.

Other Raman signals occurring in the assessed cocoa liquor spectra and related to the fatty acid fractions include the band at 1664 cm^−1^, which can be assigned to the stretching of the C=C double bonds, and the band at 1747 cm^−1^, which is related to the elongation of the C=O belonging to the ester carbonyl stretching mode [44]. Both spectral features in the spectrum of cocoa liquor seem to be typical of unsaturated fatty components, particularly the oleic acid moiety (1659 and 1744 cm^−1^) [50]. Therefore, these bands could be attributed to the oleic acid moiety from the cocoa liquor. However, the presence of other TAGs cannot be ruled out, because the signals reported previously for other TAGs are very close (1743 cm^−1^ for tripalmitin, and 1742 cm^−1^ for trimyristin and trilaurin) [51].

The previous assignment of the C=O and C=C double bonds is consistent with another work on cocoa butter that depicted peaks at 1745.4 and 1733.8 cm^−1^, representing the crystallization forms III and IV, respectively [52]. These forms correspond to the region described for the stretching vibrations due to C=O bonds. Likewise, the C=C stretching region exhibits a band at 1662.7 cm^−1^, associated with the presence of oleic acid and representative of form-IV crystallization in cocoa butter at room temperature [52]. In addition, the literature on Raman spectra reports their corresponding monoacid triglyceride assignments of C=O stretching vibrations at 1720–1750 cm^−1^, C=C stretching vibrations at 1600–1680 cm^−1^, CH_2_ or CH_3_ group scissoring deformations at 1400–1500 cm^−1^, CH_2_ twisting at approx. 1300 cm^−1^, and C–C stretching vibrations in the 1060–1090 and 1110–1180 cm^−1^ regions [48].

Although most of the predominant Raman features in the cocoa liquor spectra correspond to the fatty components, peaks from other compounds present in cocoa liquor may appear. Nonetheless, some signals that were previously assigned to the fatty fraction may overlap with others corresponding to lignin, cellulose, hemicellulose, pectins, phenolic compounds, and alkaloids, which are present in cocoa liquor in lower amounts. In this regard, the weak signals corresponding to lignin may occur in cocoa liquor’s Raman spectra. This is why a previous work reported values of the lipid–lignin ratio in the cocoa nibs [32]. The lignin features observed in the spectra that could correspond to this component appeared at 356, 456, 539, 626, 779, 1135, 1220, 1301, 1362, 1445, 1603, and 1659 cm^−1^. Consistently, the bands at similar wavelengths in the native lignin spectra have been identified at 357, 463, 537, 634, 787, 1134, 1216, 1297, 1454, 1602, and 1658 cm^−1^ [53].

Most of the strongest cellulose bands may not be visible in the cocoa liquor samples due to the overlap of more intense ones corresponding to other compounds; for instance, the reported cellulose and hemicellulose bands are expected to overlap with those of the monolignol units of lignin [54]. However, some weak signals appear in the spectra at 356, 456, 989, 1071, 1108, 1250, 1301, 1362, and 1445 cm^−1^, which are similar to those reported for softwood cellulose at 351, 458, 1073, and 1298 cm^−1^ [55]. Another study found similar wavelengths for some bands occurring in cellulose I from bacterial origin [56] and *Valonia ventricosa*’s cellulose [57]. It distinguished bands at 366 and 462 (assignment not reported), 997 and 1071 (assigned to C–O stretching ring modes), 1249 (assignment not reported), 1293 (CH_2_ twisting), 1359 (C–H deformation), and 1454 (O–H deformation) cm^−1^ [57]. Considering that hemicellulose has similar chemical bonds to cellulose, their bands could be overlapped [53].

Pectins are plant cell wall polysaccharides that are formed by D-galacturonic acid [58]. The polygalacturonic (pectic) acid spectra depicted bands at 537, 621, and 775 (ring “breezing”), 853 (C6–C5–O5–C1–O1), 990 (γ(COOH)dimers), 1079 (v(CO) + δ(OH)), 1105 (v(CC)(CO)), 1145 (v(COC) glycosidic bond, ring), 1254 (δ(CH)), and 1740 cm^−1^ (v(C=O)COOH) [59]. The cocoa liquor spectra showed bands in similar locations to those previously reported at 539, 626, 779, 860, 989, 1071, 1108, 1135, 1250, and 1747 cm^−1^.

Regarding polyphenolic compounds, cocoa and chocolate have great contents of procyanidin flavonoids, catechin, and epicatechin [60]. Cocoa liquor may show some Raman features that support their presence, as discussed below based on the occurring peaks reported in the literature. Examples of these compounds include the aromatic ring vibrations from 1300 to 1600 cm^−1^, C–C ring vibrations from 1500 to 1600 cm^−1^ due to the stretching vibration of the two opposite benzene ring quadrants and the simultaneous contraction of other quadrants, and the bending in the C–H plane. Moreover, the bands at 1400 to 1500 cm^−1^ are due to the C–C ring vibrations originating from the stretching of the ring’s semicircle, the contraction of other rings, and the simultaneous CH bending [61,62]. In this sense, the band observed at 1603 cm^−1^ in the spectra could be linked to the vibrations originating from the aromatic ring ν(C=C) of the polyphenolic compounds [63].

Methylxanthines are the most abundant alkaloids in cocoa, especially theobromine, caffeine, and theophylline; together with polyphenols, they are responsible for the beans’ characteristic bitter taste [64,65,66]. The molecular structure similarities are shown in their Raman features, mainly in the 900 to 3200 cm^−1^ range [67]. Nonetheless, the representative bands associated with alkaloids that were observed in the cocoa liquor spectra will be discussed next, even though most of them might be overlapped with others with more intense features. The theobromine spectra depicted bands at 460, 620, 777, 946, 1298, 1360, 1594, and 1660 cm^−1^ [68,69]. Similar bands with slight shifts that corroborated the presence of this alkaloid in the analyzed samples were observed at 456, 626, 779, 940, 1301, 1362, 1603, and 1659 cm^−1^; these could be assigned to δ(pyrimidine ring) + δ(CNO) + δ(CH) [69], C=C–C deformation [50], O=C–C deformation, ρ(CH_3_), ν(C–N) + ρ(CH_3_),ν(C=N) + ν(C–N), ν(C=C) + ν(C–N) + δ(CH_3_), and C=O asymmetric stretching [68,69], respectively. Consistently, a study conducted on cocoa beans and their extracts reported some of the aforementioned Raman bands at 459, 620, 776, 1296, and 1594 cm^−1^ and suggested that they constitute useful information to distinguish theobromine from theophylline [50,68]. All bands above 1000 cm^−1^ may be overlapped with more intense ones, like those reported above. The Raman spectra of caffeine depict bands at 225, 850, 975, 1080, 1131, 1188, 1241, 1600, 1656, and 700 cm^−1^ [68], while the cocoa liquor spectra of our study showed similar bands with slight shifts; this corroborates the presence of the aforementioned alkaloid in the analyzed samples. The bands at 225, 860, 1071, 1135, 1250, and 1699 cm^−1^ found in cocoa liquor may be assigned to N–C–N deformation, N=C–H deformation, C–N symmetric stretching, C–N stretching, C–N stretching, and C=N stretching, respectively [68]. Also, the bands observed at 1178, 1603, and 1659 cm^−1^ may be attributed to the C–C stretching, C=C stretching, and asymmetric C=O vibrations, respectively [68].

The presence of organic acids in Ecuadorian cocoa liquor has also been evidenced elsewhere [70]. The Raman spectra for ascorbic acid show bands at 452 (C–O in-plane deformation), 621 (OH out-of-plane deformation/C=C ring stretching), 984 (C–H and O–H bending), 1081 (C–O–C stretching and C–O–H bending), 1113 (C–O–C stretching), 1258 (C–O–H bending/twisting), 1371 (CH_2_ wagging, C–O–H bending), 1452 (CH bending, CH_2_ scissoring), and 1661 (C=C ring stretching) cm^−1^ [71,72]. The citric acid features may be present in the evaluated cocoa liquor spectra at 456, 626, 989, 1071, 1108, 1250, 1362, 1445, and 1659 cm^−1^.

**Table 1:** Experimental frequencies (cm^−1^) and assignment of the Raman spectra for the Ecuadorian cocoa liquor (CCN-51 variety).

Experimental Wavenumber (cm^−1^)	Intensity	Assignment Vibrational Modes	Proposed Compound	Reported Wavenumber (cm^−1^)	Reference
626	VW	Skeletal deformation of aromatic rings, substituent groups, and side chains	Native lignin	634	[53]
		-	Polygalacturonic (pectic) acid H–Pec	621	[59]
		C=C–C deformation	Theobromine	626	[50]
		OH out-of-plane deformation/v(C=C) ring	Ascorbic acid	621	[71,72]
779	VW	Skeletal deformation of aromatic rings, substituent groups, and side chains	Native lignin	787	[53]
		Ring “breezing”	Polygalacturonic (pectic) acid H–Pec	775	[59]
		O=C–C deformation [68]	Theobromine	777 [69]	[68,69]
860	VW	(C6–C5–O5–C1–O1)	Polygalacturonic (pectic) acid H–Pec (a-glycosidic bonds in H–Pec)	853	[59]
		N=C–H deformation	Caffeine	850	[68]
1071	M	v(C–C)	C–C in cocoa butter	1000–1150	[44]
		v(C–C)	Cocoa butter polymorphs form V	1063	[45]
		v(C–O) ring modes	Cell wall of *Valonia ventricosa* cellulose	1071	[57]
		v(CO) + δ(OH)	Polygalacturonic (pectic) acid H–Pec	1079	[59]
		$v_{s}$(C–N)	Caffeine	1080	[68]
		v(C–O–C) and δ(C-O-H)	Ascorbic acid	1081	[71,72]
1135	sh	A mode of coniferaldehyde unit	Native lignin	1134	[53]
		v(COC) glycosidic bond, ring	Polygalacturonic (pectic) acid H–Pec	1145	[59]
		v(C–N)	Caffeine	1131	[68]
1301	S	τ(CH2)	Cocoa butter polymorphs form V	1297	[45]
		τ(C–H)	C–H in cocoa butter	1200–1400	[44]
		Aryl-O of aryl-OH and aryl-O-CH3 and C=C stretching of coniferyl alcohol units	Native lignin	1297	[53]
		δ(HCC) and δ(HCO)	Softwood cellulose	1298	[55]
		τ(CH2)	Cell wall of *Valonia ventricosa* cellulose	1293	[57]
		v(C–N) + ρ(CH3)	Theobromine	1298	[69]
		v(C–N)	Theobromine	1296	[50,68]
		v(C=N) + v(C–N)	Theobromine	1360	[69]
1362	VW	C–H deformation	Cell wall of *Valonia ventricosa* cellulose	1359	[57]
		w(CH2), δ(C–O–H)	Ascorbic acid	1371	[71,72]
		δ(C–H)	C–H in cocoa butter	1400–1500	[44]
		δ(C–H)	TAGs: tripalmitin, trimyristin, trilaurin, triundecanin, and triacetin	1445	[46,47]
1445	VS	Guaiacyl ring vibration	Native lignin	1454	[53]
		O–H deformation	Cell wall of *Valonia ventricosa* cellulose	1454	[57]
		δ(CH), CH2 scissoring	Ascorbic acid	1452	[71,72]
1603	W	Symmetric aryl-ring stretching	Native lignin	1602	[53]
		v(C=C)	Aromatic ring from polyphenolic compounds	max. 1613	[63]
		v(C=C) + v(C–N) + δ (CH3)	Theobromine	1594	[69]
		v(C=C)	Caffeine	1600	[68]
1659		v(C=C)	Cocoa butter region attributed to the olefinic band	1600–1700	[44]
		Ring-conjugated v(C=C) of coniferaldehyde	Native lignin	1658	[53]
	VW	$v_{as}$ (C=O)	Theobromine	1660	[68]
		$v_{as}$ (C=O)	Caffeine	1656	[68]
		v(C=C) ring stretching	Ascorbic acid	1661	[71,72]
1747	VW	v(C=O)	C=O in cocoa butter	1700–1800	[44]
		v(C=O)COOH	Polygalacturonic (pectic) acid H-Pec	1740	[59]

v: stretching; $v_{as}\;$: asymmetric stretching; $v_{s}$: symmetric stretching; δ: bending; *w*: wagging; τ: twisting.

**Figure 2:**
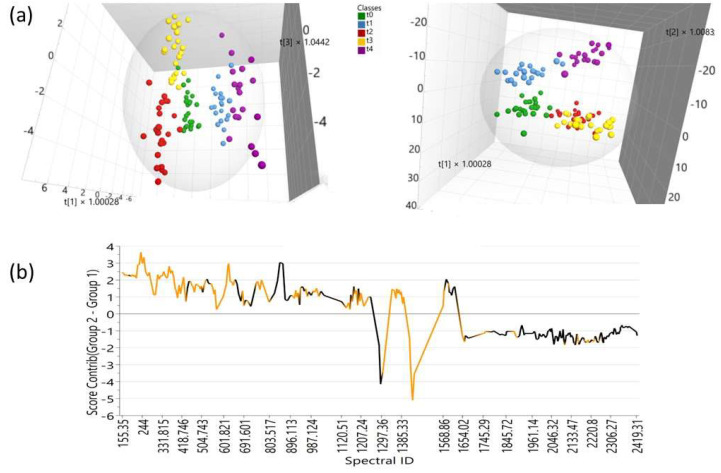
Chemometric analysis of the cocoa liquor produced using fermentation under induced magnetic fields: (**a**) Front and top (right-wise rotated) views of the 3D scatterplot for the scores. The magnetic field is shown as t0 (0 mT), t1 (5 mT), t2 (48 mT), t3 (62 mT), and t4 (80 mT). (**b**) Contribution plot for the t0 samples (lower/positive side) with respect to the t4 samples (upper/negative side). The orange-colored variables are outside the 3 std dev limit range.

On the other hand, Figure 2a shows a 3D scatterplot of the scores for the cocoa liquor produced using fermentation under low-intensity induced magnetic fields. At first glance, the data were rather scattered, especially for the samples that received large magnetic field doses (Figure 2; front location in the front view, or right-hand location in the top view). Although the lab conditions were as controlled as possible during the processing of the cocoa liquor, such data dispersion was probably due to the many variables involved in this experimental (non-commercial) processing. Despite that, the figure shows a clear discrimination of the samples with respect to the different magnetic field treatments that they received. It should be noted that the points corresponding to the largest magnetic field doses tend to be the most separate from one another. This indicates that those samples were different in their Raman responses—that is, in their composition and possible properties. Figure 2b shows the contribution plot for the t0 samples (lower part) with respect to the t4 samples (upper part) shown in Figure 2a. This plot shows the variables explaining why the t0 samples deviate from the t4 samples. The intensity of the deviation is shown as lines. This way, the larger the line values (on the Y axis), the greater the variable’s importance, and the sign of the line sequence (down = minus; up = plus) indicates the direction in which the variables deviate. The variables colored in orange are outside the three standard deviations (std dev) limit range; this means that they are even more important variables.

**Figure 3:**
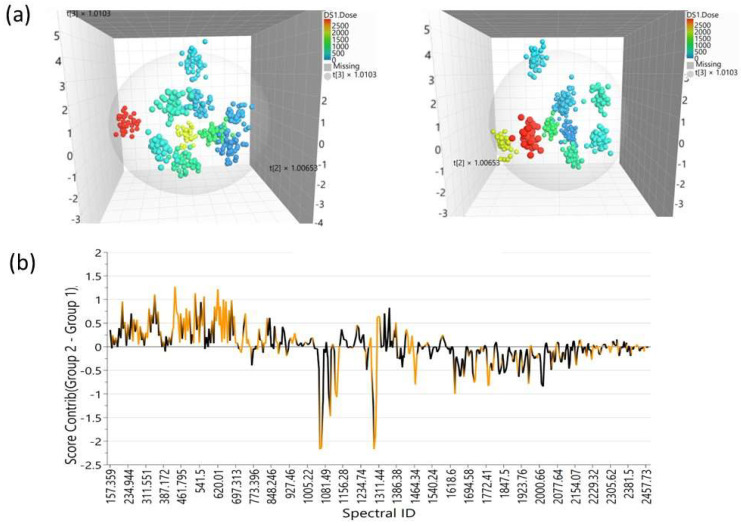
Chemometric analysis of the cocoa liquor produced after irradiating the CCN-51 samples: (**a**) Front and right-rotated side views of the 3D scatterplot for the scores. The nominal radiation doses were 0.00, 0.10, 0.20, 0.30, 0.45, 0.60, 0.75, 1.00, 2.00, and 3.00 kGy. (**b**) Contribution plot for the non-irradiated samples (lower/positive side) with respect to the 2.00 kGy irradiated samples (upper/negative side). The orange-colored variables are outside the 3 std dev limit range.

Figure 3a shows the 3D scatterplot of the scores for the cocoa liquor produced after the irradiation of the CCN-51 cocoa bean samples. These data were less scattered when compared to the set in Figure 2. The figure shows very good discrimination of the samples with respect to the different irradiation doses that they received, especially when the doses were higher. The front view (Figure 3a, left) shows that the samples treated with lower doses (darker blue dots) remained rather close to one another, which would indicate that those samples were less different from one another. This, in turn, would imply that their Raman responses were still similar at the macro scale, which suggests that their composition and possibly their properties were also somehow similar at the macro scale. On the other hand, the two largest doses (yellow and red dots on the right in Figure 3a, respectively) were completely separated from the rest of the samples and one another. This indicates that those samples were very different in their Raman responses and, thus, in their composition, and surely in their properties. Figure 3b shows the contribution plot for the non-irradiated samples (lower part) with respect to the 2.0 kGy irradiated samples (upper part), shown as the darkest blue and yellow dots in Figure 3, respectively. This plot shows the variables explaining why the non-irradiated samples deviate from the 2.0 kGy irradiated samples.

### 3.2. Sensory Attributes of the Cocoa Liquor from the Gamma-Irradiated Cocoa Beans

**Figure 4:**
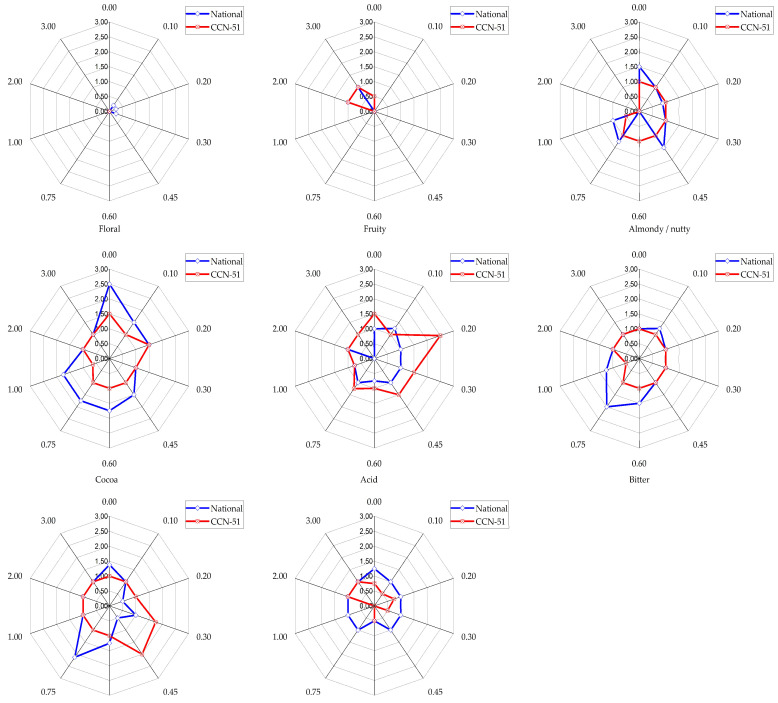
Sensory attributes of the cocoa liquor from the gamma-irradiated cocoa beans. The scales of the attributes are based on the intensity that was generated after the treatments. The radiation treatments are represented in kGy on the radial axis, where 0 corresponds to the control (non-irradiated).

Table S3 and Figure 4 show the averages of the evaluated attributes in the cocoa liquor prepared from the irradiated beans of the CCN-51 and National varieties.

There are several odor-active compounds that enable humans to perceive the characteristic chocolate flavor [73]. Cocoa presents basic flavors like acid, bitter, astringent, sweet, and salty, as well as specific flavors such as cocoa, floral, fruity, and nutty. Chocolatiers appreciate and look for attributes such as floral, cocoa, sweet, and nutty, since they give chocolate special notes [74].

The sensory attributes fruity, almondy or nutty, bitter, and astringent flavors, as well as the aroma intenseness, exhibited a very mild intensity in the CCN-51 control sample. Additionally, the floral attribute was absent, while cocoa and acid flavors showed a mild intensity. On the other hand, the National liquor control sample exhibited a medium intensity in the cocoa attribute and a mild intensity in the almondy or nutty flavor. Also, the fruity and floral attributes were absent, whereas the acid, bitter, and astringent flavors and the aroma intenseness showed a very mild intensity. The National variety showed higher values for the almondy, cocoa, and astringent flavors, as well as for the aroma intenseness.

Interestingly, the evaluated attributes in both cocoa varieties exhibited several changes in the irradiated samples. The very mild fruity attribute of the CCN-51 variety increased at 2.0 and 3.0 kGy. Therefore, it can be inferred that radiation could enhance the compounds responsible for the perception of this attribute, which was found in the fine varieties but minimal in the CCN-51 type. For example, β-myrcene and β-cis-ocimene are compounds attributed to the fruity flavor and floral aroma, respectively. β-Myrcene (5.70 ± 1.00%) and β-cis-ocimene (5.20 ± 0.70%) are present in the pulp of the fine cocoa variety SCA6, whereas the CCN-51 variety has less than 0.30% of these terpenes [75]. Other volatile compounds that have been found in CCN-51 include 2,3-butanediol, which is related to aroma, and acetophenone and 1-phenyl-2-ethanol, which are linked to fruity and floral notes [76]. The fruity and floral attributes associated with the National cocoa variety were enhanced by the irradiation, since it made them more evident to the senses. This may be akin to the enhancement in ethyl ethanoate (fruit fragrance) in rice wine achieved with the increase in the radiation dose [77].

The almondy flavor in CCN-51 was maintained with irradiation at doses up to 0.75 kGy, while at higher doses it experienced a progressive decrease. In the National variety, this flavor decreased non-uniformly with ionization at different doses, except at 0.45 kGy, where the initial value was maintained. There was a complete loss of the almondy attribute at 2.0 and 3.0 kGy in both varieties. Concerning the cocoa attribute in CCN-51, it diminished at all doses except at 0.20 kGy, where the mild intensity remained similar to the control, while the greatest loss occurred at 1.0 kGy. In the National samples, this attribute also decreased, reaching the lowest values at 0.3, 2.0, and 3.0 kGy. In cocoa and chocolate, pyrazines, aldehydes, and esters are related to the nutty, cocoa aroma, and fruity flavors, respectively. Several aroma-active compounds identified in a cocoa liquor study included tetramethylpyrazine, benzaldehyde, and 3,5-diethyl-2-methylpyrazine, which confer nutty, almondy, and cocoa aromas, respectively [78]. Thus, we can infer an influence of radiation on the compounds responsible for the aforementioned attributes.

The mild acid taste of CCN-51 was maintained at 0.3 and 0.45 kGy, while it decreased at 0.6 kGy and higher doses. The largest reduction in this attribute occurred at 0.75 kGy, and an increase was observed at 0.2 kGy. The acid taste of the National variety is lower; this behavior was maintained at almost all of the irradiation doses. This attribute increased at 0.1 kGy, but decreased at 0.6 and 1.0, and was completely lost at 3.0 kGy. Acids formed during cocoa fermentation, such as lactic and acetic acids, provide acid notes that constitute a typical flavor attribute [75,79]. Based on experimental data, it can be deduced that the gamma irradiation could decrease the acidity of both varieties at 1.0 kGy and higher doses, and to a greater extent in the CCN-51 variety. The increase in acidity could be due to the degradation of large fat molecules caused by the radiation, which renders the production of smaller ones, including free fatty acids [80]. On the other hand, the reduction in acidity might be a consequence of the hydrolytic decomposition of the acid groups caused by the gamma rays [81].

The behavior of bitter and astringent attributes could be explained by the changes in the contents of antioxidants that may affect the sensory profile of the cocoa liquor prepared from irradiated beans, since plants and plant materials use their antioxidant system as a defense mechanism against the stress caused by products of oxidative reactions, such as the free radicals released through gamma irradiation [82].

The mild bitter taste determined in the CCN-51 control sample was maintained under the different treatments and decreased only at 1.00 kGy. In contrast, this attribute in the National variety increased at 0.1, 0.6, 0.75, and 1.0 kGy. The most remarkable increment was at 0.75 kGy, which may suggest that radiation also interacts with the compounds responsible for bitter taste. The presence of methylxanthines—namely, theobromine and caffeine—provides the bitter taste that consumers perceive in Amazonian cocoa and chocolate [83]. Indeed, alkaloid and flavonoid contents, antioxidant activity, and phenolic compounds have been found to be increased in gamma-irradiated seeds [84,85]. Moreover, a correlation between total polyphenol content and bitter flavor has been evidenced [86]. This may explain the increasing bitter taste in the cocoa liquor obtained from the irradiated beans. Conversely, a study of gamma-irradiated monsooned coffee suggested that the dose produced an increasing degradation of the chlorogenic acid [87], which may explain the reduction in the bitter taste in the evaluated cocoa liquors.

The astringency in CCN-51 only changed at 0.3 and 0.45 kGy—doses at which the attribute increased. In the National variety, it decreased in all of the treatments, except at 0.75 kGy. The reduction in the cocoa beans’ astringency could improve their palatability, since an increase in bitterness, astringency, and acidity (due to inappropriate fermentation) could negatively affect the cocoa flavor and other attributes [74].

The aroma intensity in CCN-51 was completely lost when doses of 0.45, 0.75, and 1.0 kGy were used, but it increased at 2.0 and 3.0 kGy. Unlike what was observed in the bulk variety, the aroma of the National variety cocoa liquor decreased in all of the radiation treatments, with a maximum reduction at 0.6 kGy.

Other authors have also found differences in the flavor profiles of irradiated samples. A study of dried scallions exposed separately to three irradiation sources and subsequently assessed by electronic sensing (e-nose and e-tongue) concluded that the irradiation treatments affected the amounts of the identified volatile compounds. A significant increase associated with the aroma profile was found at 4 and 7 kGy, while the perception of the taste attributes increased at the last dose (7 kGy) for the samples irradiated by an e-beam and with gamma rays. There was a detectable change in the volatile compound profile during the gamma-ray treatment [88]. Kakdugi samples prepared with red pepper powder ionized with gamma rays at doses of 0 (control), 3, 5, and 7 kGy were grouped in four different clusters, after the e-nose analysis at the beginning of the fermentation process. This could have been due to the influence of radiation on the odor molecules [89]. Grapes subjected to gamma irradiation at 0.67, 1.3, 2.0, and 2.7 kGy exhibited increases in their anthocyanin concentrations at higher doses, whilst maintaining their flavanol and flavonol contents. The concentrations of the aromatic compounds associated with the fruity and floral notes showed an increase at the first three doses [90].

The increase in the perception of aroma attributes may be explained by the influence of radiation on the compound bonds. For instance, bounded glycosides in grapevines were identified as odorless, in contrast with free volatile compounds, which showed an impact on the aroma and flavor [90,91]. Moreover, a study on irradiated nutmeg concluded that glycosides responsible for aroma broke down due to the irradiation. Consequently, there was an enhancement in the contents of the nutmeg’s volatile compounds [90,92].

Both the CCN-51 and National varieties showed a rancid flavor in the samples irradiated at 1.0 kGy, and it was more intense at 2.0 and 3.0 kGy. This could be attributed to the interaction of the triacylglycerol fatty acids with gamma rays; in particular, unsaturated fatty acids are susceptible to radiation, and they generate products that contribute to rancidity [93].

## 4. Conclusions

To the best of our knowledge, this study is the first to assess the influence of induced magnetic fields during cocoa beans’ fermentation on the constituents of the cocoa liquor obtained from them, as well as the influence of gamma irradiation of fermented cocoa beans on the flavor constituents of the liquor. Raman spectroscopy enables the identification of the main compounds that are present in cocoa liquor. The Raman spectra of cocoa liquor were attributed to lipids, lignocellulosic compounds, pectins, flavonoids, theobromine, and organic acids. Also, the irradiation dose, the electric field intensity, and the cocoa variety could be discriminated using Raman spectra and chemometric tools. Therefore, this study provides evidence for the potential application of this spectroscopic tool to identify and evaluate irradiated foods and fermented products exposed under induced magnetic fields. The cocoa constituents exhibited interactions with gamma rays that could be translated into changes in the sensory profile of the resulting cocoa liquors, which are perceptible to human senses. In this regard, their sensory attributes exhibited varying behaviors depending on the dose, and some of them showed a marked tendency (i.e., rancid) at 1 kGy and higher doses. Indeed, radiation exerts an influence on the cocoa bean constituents and the compounds responsible for the sensory attributes in the cocoa liquor prepared with irradiated National and CCN-51 cocoa beans. The determination of a dose that provides the benefits of irradiation while preserving cocoa’s nutritional and flavor attributes should be pursued in future research.

Regarding the fermentation of the cocoa under induced magnetic fields, further experiments are needed to find out and better explain all of the variables responsible for the data dispersion, which would help improve the cocoa liquor processing.

## Data Availability

The data used to support the findings of this study can be made available by the corresponding author upon request.

## Supplementary Materials

The following supporting information can be downloaded at: https://www.mdpi.com/article/10.3390/foods12213924/s1, Figure S1: Schematic representation of the dosimeter location at four positions in the plastic trays, identification of the maximum and minimum dose, and dose uniformity rate (DUR) calculus at each irradiation treatment in the (a) National and (b) CCN-51 varieties. Figure S2: Location of the four dosimeters at positions A, B, C, and D in the plastic tray containing the cocoa beans for each treatment. Table S1: Time treatment, nominal dose, dosimeter readings, and DUR for the irradiated National cocoa beans. Table S2: Time treatment, nominal dose, dosimeter readings, and DUR for the irradiated CCN-51 cocoa beans. Table S3: Averages of the evaluated attributes in the cocoa liquor obtained from fermented and dried beans irradiated at different nominal doses for the CCN-51 and National varieties.
