# Supplementary material for: Raman Spectroscopic and Sensory Evaluation of Cocoa Liquor Prepared with Ecuadorian Cocoa Beans Treated with Gamma Irradiation or Induced Electromagnetic Field Fermentation
Source: Foods. 2023 Oct 26;12(21):3924. doi: 10.3390/foods12213924 (PMC10647436; doi:10.3390/foods12213924)
Supplement: Supplementary file 1 [file foods-12-03924-s001.zip › foods-2607990-supplementary.pdf]

Article

Raman spectroscopic and sensory evaluation of cocoa liquor prepared with Ecuadorian cocoa beans treated with gamma irradiation or induced electromagnetic field fermentation.

Tania María Guzmán-Armenteros [1], Jenny Ruales[1], Cristina Cuesta-Plúa[2], Juan Bravo[2], Marco Sinche[3], Edwin Vera[1], Edison Vera[3], Paul Vargas[3*], Valerian Ciobota[4], Fernando Ortega[5,6], Andrés Proaño[7], Armando Echeverría[8], and Luis Ramos-Guerrero[9*]

Supplementary Material

Table S1. Time treatment, nominal dose, dosimeter readings, and DUR for the irradiated National cocoa beans.

Variety	EPR	Irradiation time (h)	Nominal Dose (kGy)	A (kGy)			B (kGy)			C (kGy)			D (kGy)			DUR
	Holder used			Dosimeter Read. 1	Dosimeter Read. 2	Mean	Dosimeter Read. 1	Dosimeter Read. 2	Mean	Dosimeter Read. 1	Dosimeter Read. 2	Mean	Dosimeter Read. 1	Dosimeter Read. 2	Mean	
National	PX063	1.22	0.10	0.10	0.10	0.10	0.11	0.11	0.11	0.11	0.11	0.11	0.09	0.09	0.09	1.22
	PX063	2.43	0.20	0.21	0.21	0.21	0.23	0.23	0.23	0.22	0.22	0.22	0.18	0.18	0.18	1.28
	PX063	3.65	0.30	0.32	0.32	0.32	0.34	0.34	0.34	0.33	0.33	0.33	0.27	0.27	0.27	1.26
	PX063	5.48	0.45	0,40	0.40	0.40	0.46	0.46	0.46	0.44	0.44	0.44	0.37	0.37	0.37	1.24
	PX063	7.30	0.60	0.63	0.63	0.63	0.67	0.67	0.67	0.65	0.65	0.65	0.53	0.53	0.53	1.26
	PX063	9.12	0.75	0.69	0.69	0.69	0.79	0.79	0.79	0.80	0.80	0.80	0.61	0.61	0.61	1.31
	PX063	12.13	1.00	1.07	1.07	1.07	1.14	1.14	1.14	1.07	1.07	1.07	0.92	0.92	0.92	1.24
	PL	24.67	2.00	2.47	2.46	2.47	2.81	2.79	2.80	2.57	2.56	2.57	2.21	2.21	2.21	1.27
	PH0156	36.93	3.00	-	-	-	3.05	3.02	3.04	2.92	2.93	2.93	-	-	-	1.23
	PL	36.93	3.00	2.75	2.76	2.76	-	-	-	-	-	-	2.49	2.47	2.48	

Table S2. Time treatment, nominal dose, dosimeter readings, and DUR for the irradiated CCN-51 cocoa beans.

Variety	EPR	Irradiation time (h)	Nominal Dose (kGy)	A (kGy)			B (kGy)			C (kGy)			D (kGy)			DUR
	Holder used			Dosimeter Mean 1	Dosimeter Read. 2	Mean	Dosimeter Read. 1	Dosimeter Read 2	Mean	Dosimeter Read. 1	Dosimeter Read. 2	Mean	Dosimeter Read. 1	Dosimeter Read. 2	Mean	
CCN-51	PX063	1.22	0.10	0.11	0.11	0.11	0.12	0.12	0.12	0.12	0.12	0.12	0.09	0.09	0.09	1.33
	PX063	2.43	0.20	0.24	0.24	0.24	0.29	0.29	0.29	0.29	0.29	0.29	0.22	0.22	0.22	1.32
	PX063	3.58	0.30	0.26	0.32	0.29	0.32	0.32	0.32	0.30	0.30	0.30	0.24	0.24	0.24	1.33
	PX063	5.47	0.45	0.48	0.48	0.48	0.54	0.54	0.54	0.52	0.52	0.52	0.41	0.41	0.41	1.32
	PX063	7.28	0.60	0.65	0.65	0.65	0.68	0.68	0.68	0.63	0.63	0.63	0.51	0.51	0.51	1.33
	PX063	9.12	0.75	0.72	0.72	0.72	0.81	0.81	0.81	0.80	0.80	0.80	0.59	0.59	0.59	1.37
	PX063	12.15	1.00	1.07	1.07	1.07	1.23	1.23	1.23	1.21	1.21	1.21	0.94	0.94	0.94	1.31
	PL	24.67	2.00	2.42	2.42	2.42	2.81	2.84	2.83	2.67	2.63	2.65	2.15	2.16	2.16	1.31
	PH0156	36.85	3.00	2.93	2.93	2.93	3.18	3.20	3.19	3.29	3.37	3.33	-	-	-	1.17
	PL	36.85	3.00	-	-	-	-	-	-	-	-	-	2.84	2.84	2.84	

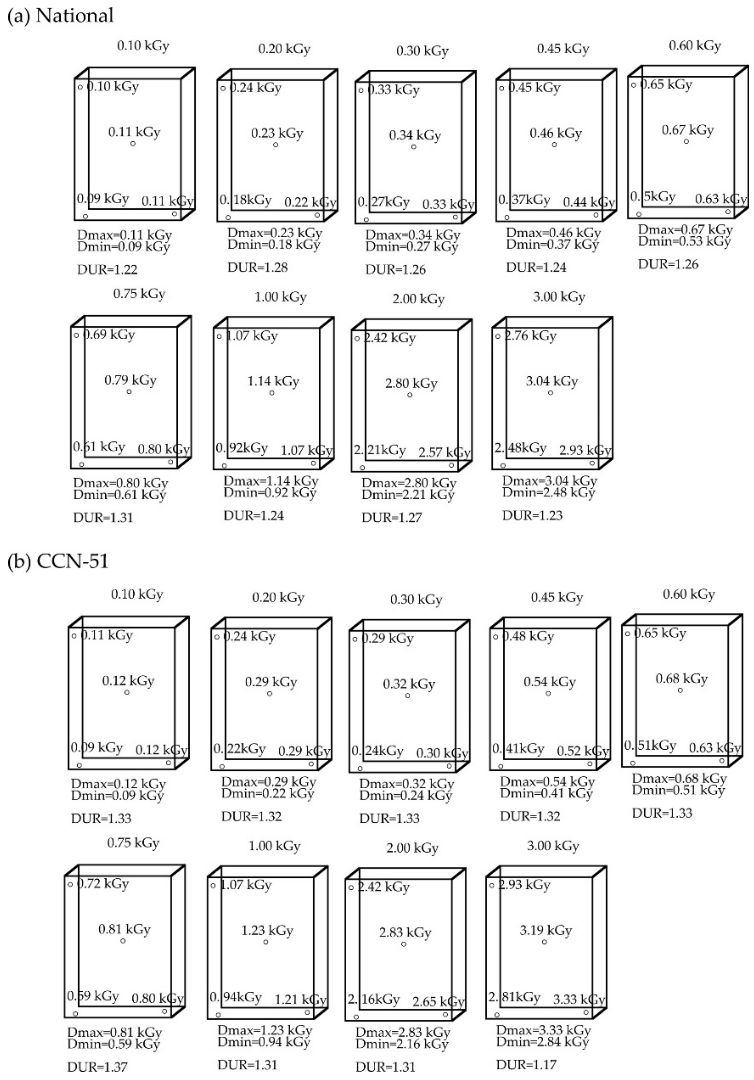

Figure S1. Schematic representation of the dosimeter location at four positions in the plastic trays, identification of the maximum and minimum dose and Dose Uniformity Rates (DUR) calculus at each irradiation treatment in varieties (a) National; (b) CCN-51.

The $DUR = \frac{D_{max}}{D_{min}}$ (1) was calculated by dividind the maximum dose to the minimum one.

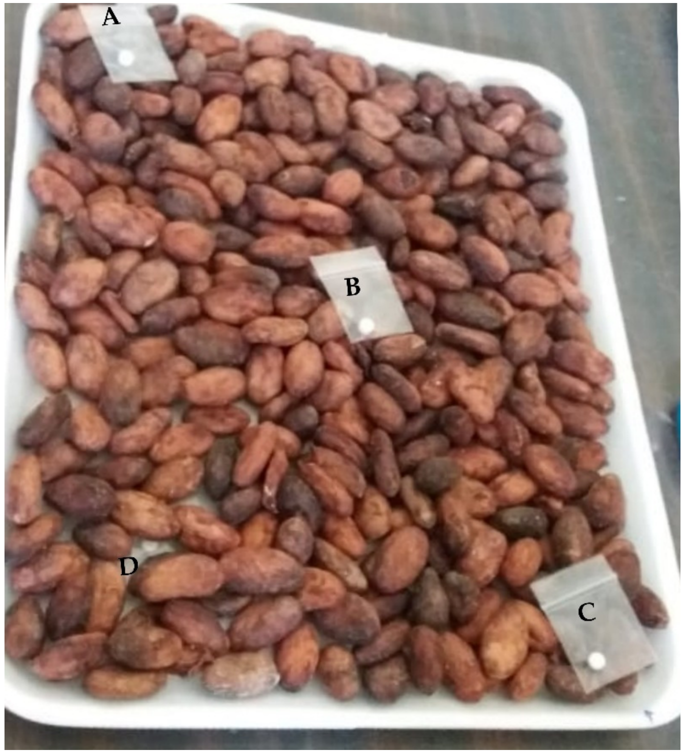

Figure S2. Location of the four dosimeters at A, B, C, and D position in the plastic tray containing the cocoa beans for each treatment.

Table S3. Averages of the evaluated attributes in the cocoa liquor obtained from fermented and dried beans irradiated at different nominal doses for the CCN-51 and National varieties.

Variety	Nominal Dose (kGy)	Floral	Fruity	Almond/nut	Cocoa	Acid	Bitter	Astringent	Aroma intensity
National	0.00	0.00 ± 0.00	0.00 ± 0.00	1.50 ± 0.71	2.50 ± 0.71	1.00 ± 0.00	1.00 ± 0.00	1.38 ± 0.53	1.25 ± 0.35
	0.10	0.25 ± 0.35	0.00 ± 0.00	1.00 ± 1.41	1.50 ± 0.71	1.25 ± 0.35	1.25 ± 0.35	1.00 ± 1.41	1.00 ± 0.00
	0.20	0.25 ± 0.35	0.00 ± 0.00	0.88 ± 0.18	1.50 ± 0.71	1.00 ± 0.00	1.00 ± 0.00	0.50 ± 0.71	1.00 ± 0.00
	0.30	0.25 ± 0.35	0.00 ± 0.00	1.00 ± 0.00	1.00 ± 0.00	1.00 ± 0.00	1.00 ± 0.00	1.00 ± 0.00	1.00 ± 0.00
	0.45	0.00 ± 0.00	0.00 ± 0.00	1.50 ± 0.71	1.50 ± 0.71	1.00 ± 0.00	1.00 ± 0.00	0.50 ± 0.71	1.00 ± 0.00
	0.60	0.00 ± 0.00	0.00 ± 0.00	0.00 ± 0.00	1.75 ± 1.06	0.75 ± 0.35	1.50 ± 0.00	1.25 ± 0.35	0.50 ± 0.71
	0.75	0.00 ± 0.00	0.00 ± 0.00	1.25 ± 0.35	1.75 ± 0.35	1.00 ± 0.71	2.00 ± 0.00	2.13 ± 0.53	1.00 ± 0.00
	1.00	0.00 ± 0.00	0.00 ± 0.00	1.00 ± 0.00	1.75 ± 1.06	0.75 ± 0.35	1.25 ± 0.35	1.00 ± 0.00	1.00 ± 0.00
	2.00	0.00 ± 0.00	1.00 ± 0.00	0.00 ± 0.00	1.00 ± 0.00	1.00 ± 0.00	1.00 ± 0.00	1.00 ± 0.00	1.00 ± 0.00
	3.00	0.00 ± 0.00	1.00 ± 0.00	0.00 ± 0.00	1.00 ± 0.00	0.00 ± 0.00	1.00 ± 0.00	1.00 ± 0.00	1.00 ± 0.00
CCN-51	0.00	0.00 ± 0.00	0.50 ± 0.71	1.00 ± 1.41	1.5 ± 0.71	1.50 ± 0.71	1.00 ± 0.00	1.00 ± 0.00	0.75 ± 0.35
	0.10	0.00 ± 0.00	0.00 ± 0.00	1.00 ± 0.00	1.00 ± 0.00	1.00 ± 0.00	1.00 ± 0.00	1.00 ± 0.00	0.50 ± 0.71
	0.20	0.00 ± 0.00	0.00 ± 0.00	1.00 ± 0.00	1.50 ± 0.71	2.50 ± 0.00	1.00 ± 0.00	1.00 ± 0.00	0.75 ± 1.06
	0.30	0.00 ± 0.00	0.00 ± 0.00	1.00 ± 0.00	1.00 ± 0.00	1.50 ± 0.71	1.00 ± 0.00	1.75 ± 1.06	0.50 ± 0.71
	0.45	0.00 ± 0.00	0.00 ± 0.00	1.00 ± 0.00	1.00 ± 0.00	1.50 ± 0.71	1.00 ± 0.00	2.00 ± 0.71	0.00 ± 0.00
	0.60	0.00 ± 0.00	0.00 ± 0.00	1.00 ± 0.00	1.00 ± 0.00	1.00 ± 0.00	1.00 ± 0.00	1.00 ± 0.00	0.50 ± 0.71
	0.75	0.00 ± 0.00	0.00 ± 0.00	1.00 ± 0.00	1.00 ± 0.00	1.25 ± 1.06	1.00 ± 0.00	1.00 ± 0.00	0.00 ± 0.00
	1.00	0.00 ± 0.00	0.00 ± 0.00	0.50 ± 0.00	0.63 ± 0.18	0.75 ± 0.35	0.50 ± 0.71	1.00 ± 1.41	0.00 ± 0.00
	2.00	0.00 ± 0.00	1.00 ± 0.00	0.00 ± 0.00	1.00 ± 0.00	1.00 ± 0.00	1.00 ± 0.00	1.00 ± 0.00	1.00 ± 0.00
	3.00	0.00 ± 0.00	1.00 ± 0.00	0.00 ± 0.00	1.00 ± 0.00	1.00 ± 0.00	1.00 ± 0.00	1.00 ± 0.00	1.00 ± 0.00

$\bar{x} \pm \sigma \ (n = 2)$
